# Using mangrove and field observation data to identify fine-scale species distributions: a case study in bockadams (Serpentes: Homalopsidae: *Cerberus*)

**DOI:** 10.1098/rsos.240483

**Published:** 2024-10-02

**Authors:** Justin M. Bernstein, Joward B. Bautista, Michael A. Clores, Rafe M. Brown, Sara Ruane, Marites B. Sanguila, Mary Grace Joyce Alis-Besenio, Cory Lyn F. Pejo, Michael A. Cuesta

**Affiliations:** ^1^ Center for Genomics, University of Kansas, 1345 Jayhawk Boulevard, Lawrence, KS 66045, USA; ^2^ Social Science Research Center, Ateneo de Naga University, Ateneo Avenue, Barangay Bagumbayan Sur, Naga City 4400, Philippines; ^3^ Partido State University, Caramoan Campus, Barangay Cadong, Caramoan, Philippines; ^4^ Biodiversity Institute and Department of Ecology and Evolutionary Biology, University of Kansas, 1345 Jayhawk Boulevard, Lawrence, KS 66045, USA; ^5^ Life Sciences Section, Negaunee Integrative Research Center, Field Museum, 1400 South Lake Shore Drive, Chicago, IL 60605, USA; ^6^ Biodiversity Informatics and Research Center, Father Saturnino Urios University, Butuan City, Agusan Del Norte 8600, Philippines

**Keywords:** biodiversity, conservation, niche models, Philippines, snakes, Southeast Asia

## Abstract

Characterization of species distributions is a fundamental challenge in biodiversity science, with particular significance for downstream evolutionary studies, conservation efforts, field-based faunal studies and estimates of species diversity. Checklists and phylogenetic studies often focus on poorly known, rare taxa with limited ranges. However, studies of widely distributed, ecologically important species that are abundant in their preferred microhabitats are also important for systematics and local conservation efforts, but less often studied. We collected novel natural history data during fieldwork (2019–2023) for Philippine populations of bockadams (Homalopsidae: *Cerberus*), one of the most abundant vertebrates in Southeast Asian aquatic systems. Considered a coastal snake, many studies report *Cerberus* inland. We report the frequency of encounters of *Cerberus schneiderii*, and the IUCN data-deficient, Philippine-endemic *Cerberus microlepis* during six expeditions (62 days; 1041 person-hours). We report new occurrence data for 69 *C. schneiderii* and *6 C. microlepis* for coastal and inland populations, water measurements and dietary observations. Regression analyses and ecological niche models show the importance of coastal and mangrove habitats for *Cerberus*. Our study is the most comprehensive assessment of Philippine *Cerberus* populations to date and provides critical baseline natural history data for downstream research on widespread and range-restricted species of Southeast Asian snakes.

## 1. Introduction

Accurate distribution and natural history data have become an integral part of biodiversity surveys and evolutionary studies. Investigations at local scales (within species’ ranges), undertaken to quantitatively assess the presence/absence of a species at a particular location (coordinate data, corresponding to individuals encountered) remain the valued, critical input data for ecological niche models and conservation efforts [1,2]. However, the accuracy and value of such models—for understanding diversity and species ranges—rely heavily on field-collected species’ occurrence data [3,4]. Consequently, these analyses provide base data not only for conservation but also for improving our understanding of niche differentiation between species and diversification scenarios in a broad array of organismal groups [5–8]. In addition to point data, natural history observations can also be used as *a priori* information in trait-based modelling analyses [9–11]. Trait-based data such as diet, behaviour of the organism and habitat preference have been used to identify repeated evolution of structures [12], provide support for alternative scenarios in speciation hypotheses [13] and broaden our understanding of the evolution of feeding and predation strategies [14,15]. Although data pertaining to species ranges and their natural history are important for evolution and conservation science, much of research has focused on poorly known, rare or narrowly distributed taxa and the challenge posed by urgent needs to improve their models and estimates of habitat suitability, but with few available data [16,17]. In contrast, widespread and abundant species may be opportune study systems for testing hypotheses of lineage diversification, population genomics and distribution modelling [18,19].

**Figure 1.**
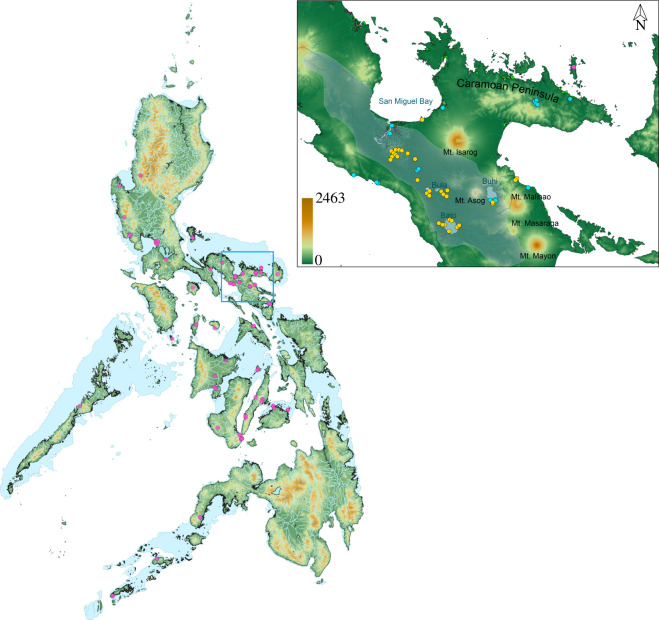
Map of The Philippines with sampling of occurrence records (purple points; point = one individual) from online repositories and field collection efforts for this study. Shaded blue regions between islands represent the Pleistocene isobath at 120 m below present-day sea level, linking the Pleistocene Aggregate Island Complexes (PAICs). Map coloured by elevation. Light blue lines within islands are river systems in The Philippines. Inset map: zoomed in view of Camarines Sur province. Bright green regions on coasts represent mangroves from Global Mangrove Watch (GMW) 2020 dataset. Blue records show collected or observed specimens from field expeditions for this study; yellow dots are visited areas from our field expeditions in 2023 that did not result in any *Cerberus* records. Blue shaded polygon in the inset map represents the Bicol River Basin (BRB). Map coloured by elevation (SRTM Tiles from NASA).

The mud snakes in the family Homalopsidae comprise a relatively small family compared to other Caenophidian groups and are represented by 60 species in 26 genera. Many of the taxa in this group differ in their microhabitat preferences of aquatic systems, tolerance to salinity, soft tissue sensory structures, predation strategies, diet preference and body size [12,14,20,21]. The evolution of homalopsids has been investigated recently with dense sampling using both molecular and morphological datasets, providing insight into Southeast Asian biogeographic paradigms [13,22] and evolution of unique oral glands in snakes [12]. Some homalopsids have markedly dense populations in Southeast Asia. *Cerberus schneiderii* (Southeast Asian dog-faced water snake; also known as the Southeast Asian bockadam), one of the most wide-ranging vertebrates in the world (figure 1), may also be the most densely populated vertebrate in some of Southeast Asia’s aquatic systems [20]. With the exception of a few studies [23–25], and despite their large range, *C. schneiderii* is a species conspicuously characterized by a lack of natural history data or local occurrence records. It is considered a coastal mangrove ‘specialist’, but reports of inland populations have been documented [20,23]. Knowledge of where this taxon is likely to occur and how often it is found in inland aquatic habitats will be useful for regional and local biodiversity surveys, as well as understanding if these piscivorous snakes play important roles for economies that rely on fish populations.

In this study, we report our distributional and natural history findings of Philippine *C. schneiderii* and *Cerberus microlepis*, the latter of which has only conclusively been recorded, far inland, in Lake Buhi, Camarines Sur Province (figure 1). In 2019, 2022 and 2023, we conducted field surveys for these aquatic species in the Bicol Faunal Region (Camarines Sur Province, Philippines; figure 1). The Bicol Region’s heterogenous topography is composed of volcanoes, mountains, mangroves, river systems and the partially isolated Caramoan Peninsula, all of which harbour great herpetofaunal diversity, some of which are still being described [26,27]. Using microhabitat focused, purposive transect sampling and opportunistic survey approaches, we report new Philippine distribution, dietary and behaviour records for *C. schneiderii* and *C. microlepis*.

## 2. Material and methods

### 2.1. Study area

Our search efforts for *Cerberus* took place in the Bicol Faunal Region of Camarines Sur Province, Philippines (southeast Luzon Island; figure 1). Camarines Sur is located at 13.6218° N, 123.1948° E (central coordinates), and is part of the Luzon PAIC. Camarines Sur receives an annual rainfall of 2565 mm, with the driest seasons occurring from March to May. The Bicol Region contains some of the lowest and highest elevations found in the Philippines. Its heterogeneous topography is characterized by a series of volcanoes that range in elevation from 1196 m (Mt Asog/Mt Iriga) to 2463 m (Mt Mayon). Our surveys took place in two subregions: the Caramoan Peninsula and the Bicol River Basin, the latter which occurs off the peninsula (figure 1).

The Caramoan Peninsula stretches out from the northeastern region of Camarines Sur (13.7472° N, 123.7652° E), which contains mountains as high as ~1000 m. Habitats on this landmass are widely variable, containing rugged hills with primary, secondary and mixed forests, limestone karst peaks and cliffs, rock outcrops, caves, gorges and low-elevational areas containing agricultural fields, plains, river valleys, riparian corridors, mangrove forests, beach forests, coastal forests and white sand beaches. The climate falls within the Philippine Climate Type-2, receiving an annual average rainfall of 3188 mm and average monthly temperatures of 25.7−28.4°C. Our efforts in this study are based in the Municipality of Caramoan.

The Bicol River Basin is a 3770 km^2^ area and represents the eighth largest river basin in The Philippines, with one of the most frequent flooding regimes. The basin’s length is juxtaposed next to the Ragay Hills in the southwest and Mt Malinao, Mt Asog (Iriga), Mt Mayon and Mt Isarog towards the northeast [28]. Rainfall in this region is constant throughout the year, with precipitation peaking from November to February; annual rainfall reaches 2000−3600 mm in the basin [29]. Two major rivers, the Bicol River and Libmanan River, empty into San Miguel Bay at the northern coasts of Camarines Sur. Our study sites along the Bicol River Basin span several municipalities that are adjacent to the basin, with the exception of two coastal localities to the west (Municipality of Pasacao) and east (Municipality of Tiwi, Albay Province). Three major lakes are found within the Bicol River Basin: Lakes Bato, Baao and Buhi (figure 1).

### 2.2. Encounter surveys and collection methods

Six separate field surveys were conducted intermittently in the Bicol River Basin and surrounding areas (BRB) and the Caramoan Peninsula (CP)—location and number of survey days and are given parenthetically (table 1): March 2019 (BRB; 2), August 2022 (CP; 9), October–November 2022 (CP; 14), December 2022 (CP; 11), March–April 2023 (BRB; 20) and June 2023 (BRB; 6). Purposive transect and opportunistic search methods were used, searching different aquatic habitat types (e.g. lakes, rivers, tributaries, small freshwater streams, mangroves, intertidal flats, open water close to coasts, fish ponds (inland), fish farms (coastal) and rice paddy fields). Rivers and streams that occurred inland were also searched, as *Cerberus* has been found as far inland as ~41 km upstream from the mouths of rivers [24]. We consider coastal, inland–coastal and inland localities to be <1, 1−5 and >5 km from the closest shoreline, respectively. During our 2023 expeditions, we recorded water temperature, specific gravity and salinity (parts per thousands; PPT) for several localities to better understand environmental qualities of the waters *Cerberus* use as habitat. Measurements were taken where the first *Cerberus* were detected for that survey day.

**Table 1.** *Cerberus* survey results from expeditions from 2019 to 2023. Location and respective number of searchers, person-hours (hours × number of searchers) and *Cerberus* found are provided.

date	time duration (24 h)	locality	municipality	province	coastal versus inland	hours searched for obtaining *Cerberus*	number of searchers	person-hours	number *Cerberus* found
4/3/2019	18.00−19.00	Barangay San Pascual, in stream ~100 ft from the mouth of Lake Buhi	Buhi	Camarines Suri	inland	1	6	6	6
16/3/2019	20.20	Barangay Ponong, in Libmanan (Bicol) River, <1 km northeast from edge of Doncillia Island	Magarao	Camarines Sur	inland–coastal	1.00	4	4	1
4/8/2022	19.00−22.00	Barangay Tabiguian	Caramoan	Camarines Sur	inland–coastal	3.00	13	39	0
4/8/2022	09.00−10.00	Barangay Tabiguian	Caramoan	Camarines Sur	inland–coastal	1.00	13	13	0
5/8/2022	19.00−23.00	Barangay Tabiguian	Caramoan	Camarines Sur	inland–coastal	4.00	13	52	0
6/8/2022	18.00−22.00	Barangay Tabiguian	Caramoan	Camarines Sur	inland–coastal	4.00	13	52	0
8/8/2022	19.00−22.00	Barangay Tabiguian	Caramoan	Camarines Sur	inland–coastal	3.00	12	36	0
9/8/2022	19.00−22.00	Barangay Santa Cruz	Caramoan	Camarines Sur	inland–coastal	3.00	12	36	1
12/8/2022	08.00−10.00	Barangay Tawog	Caramoan	Camarines Sur	inland–coastal	2.00	12	24	0
12/8/2022	19.00−22.00	Barangay Tawog	Caramoan	Camarines Sur	inland–coastal	4.00	12	48	0
13/8/2022	19.00−22.00	Barangay Tawog	Caramoan	Camarines Sur	inland–coastal	4.00	12	48	0
18/10/2022	19.00−21.00	Barangay Ilawod, Sitio Cabotonan	Caramoan	Camarines Sur	inland–coastal	3.00	8	24	0
19/10/2022	08.00−09.00	Barangay Ilawod, Sitio Cabotonan	Caramoan	Camarines Sur	inland–coastal	1.00	8	8	0
19/10/2022	14.00−16.30	Barangay Ilawod, Sitio Cabotonan	Caramoan	Camarines Sur	inland–coastal	2.50	8	20	0
19/10/2022	18.30−22.30	Barangay Ilawod, Sitio Cabotonan	Caramoan	Camarines Sur	inland–coastal	4.00	8	32	0
20/10/2022	19.00−21.00	Barangay Ilawod, Sitio Cabotonan	Caramoan	Camarines Sur	inland–coastal	3.00	8	24	0
21/10/2022	20.00−22.00	Barangay Ilawod, Sitio Cabotonan	Caramoan	Camarines Sur	inland–coastal	2.00	8	16	0
22/10/2022	18.00−22.00	Barangay Ilawod, Sitio Cabotonan	Caramoan	Camarines Sur	inland–coastal	4.00	8	32	0
23/10/2022	17.30−20.30	Barangay Ilawod, Sitio Cabotonan	Caramoan	Camarines Sur	inland–coastal	4.00	8	32	0
24/10/2022	17.30−02.30	Barangay Ilawod, Sitio Cabotonan	Caramoan	Camarines Sur	inland–coastal	5.00	8	40	0
25/10/2022	09.00−10.00	Barangay Ilawod, Sitio Cabotonan	Caramoan	Camarines Sur	inland–coastal	1.00	8	8	0
25/10/2022	17.00−22.00	Barangay Ilawod, Sitio Cabotonan	Caramoan	Camarines Sur	inland–coastal	5.00	8	40	0
30/10/2022	19.00−21.00	Barangay Ilawod, Sitio Cabotonan	Caramoan	Camarines Sur	inland–coastal	2.00	8	16	0
31/10/2022	19.00−22.00	Barangay Ilawod, Sitio Cabotonan	Caramoan	Camarines Sur	inland–coastal	3.00	8	24	0
1/11/2022	18.00−22.00	Barangay Ilawod, Tayak Lake	Caramoan	Camarines Sur	coastal	4.00	8	32	0
1/12/2022	14.00−15.15	Barangay Ilawod, Sitio Cabudian, eastern boundary of Caramoan National Park	Caramoan	Camarines Sur	inland–coastal	0.75	5	3.75	0
1/12/2022	18.00−21.30	Barangay Ilawod, Sitio Cabudian, eastern boundary of Caramoan National Park	Caramoan	Camarines Sur	inland–coastal	3.50	5	17.5	0
2/12/2022	14.00−15.00	Barangay Ilawod, Sitio Cabudian, eastern boundary of Caramoan National Park	Caramoan	Camarines Sur	inland–coastal	1.00	5	5	0
2/12/2022	18.00−19.30	Barangay Ilawod, Sitio Cabudian, eastern boundary of Caramoan National Park	Caramoan	Camarines Sur	inland–coastal	1.50	5	7.5	0
3/12/2022	09.00−10.00	Barangay Ilawod, Sitio Cabudian, eastern boundary of Caramoan National Park	Caramoan	Camarines Sur	inland–coastal	1.00	5	5	0
3/12/2022	18.30−23.00	Barangay Ilawod, Sitio Cabudian, eastern boundary of Caramoan National Park	Caramoan	Camarines Sur	inland–coastal	4.50	5	22.5	0
4/12/2022	19.00−20.00	Barangay Ilawod, Sitio Cabudian, eastern boundary of Caramoan National Park	Caramoan	Camarines Sur	inland–coastal	1.00	5	5	0
5/12/2022	17.30−20.00	Barangay Ilawod, Sitio Cabudian, eastern boundary of Caramoan National Park	Caramoan	Camarines Sur	inland–coastal	2.50	5	12.5	0
7/12/2022	09.00−10.00	Barangay Ilawod, Sitio Cabudian, eastern boundary of Caramoan National Park	Caramoan	Camarines Sur	inland–coastal	1	5	5	0
7/12/2022	17.30−23.00	Barangay Ilawod, Sitio Cabudian, eastern boundary of Caramoan National Park	Caramoan	Camarines Sur	inland–coastal	5.5	5	27.5	0
8/12/2022	08.30−09.30	Barangay Ilawod, Sitio Cabudian, eastern boundary of Caramoan National Park	Caramoan	Camarines Sur	inland–coastal	1	5	5	0
17/3/2023	18.00−20.00	Zone 7, Barangay Balongay, in shallow water on top of mud in mangrove edge or in Libmanan River	Calabanga	Camarines Sur	coastal	2	5	10	24
17/3/2023	20.00−21.00	Barangay Cagsao, up against the Cabanbanan mangroves	Calabanga	Camarines Sur	coastal	0.25	4	1	0
17/3/2023	20.00−21.00	Barangay Belen	Calabanga	Camarines Sur	coastal	0.25	4	1	0
18/3/2023	21.30	Barangay Ponong, in Libmanan (Bicol) River tributary, <1 km northeast from edge of Doncillia Island	Magarao	Camarines Sur	coastal	0.5	8	4	1
19/3/2023	19.00−20.30	Zone 1, Barangay Taban	Minalabac	Camarines Sur	inland	1.5	6	9	0
20/3/2023	18.00−00.00	Bicol River	Minalabac	Camarines Sur	inland	8	6	48	0
21/3/2023	19.00−20.30	Barangay Santo Niño	Minalabac	Camarines Sur	inland	1.5	6	9	0
21/3/2023	19.00−20.30	Barangay Santo Niño	Minalabac	Camarines Sur	inland	1.5	6	9	0
22/3/2023	19.00−20.30	Lake Baao and Baao Water District	Baao	Camarines Sur	inland	1.5	6	9	0
23/3/2023	19.00−21.00	Waras River, Barangay San Francisco	Nabua	Camarines Sur	inland	2	6	12	0
24/3/2023	19.00−21.30	Agos River, Lake Bato	Bato	Camarines Sur	inland	2.5	6	15	0
25/3/2023	19.00−21.00	Lake Bato	Bato	Camarines Sur	inland	2	6	12	0
28/3/2023	18.00−21.00	Sowong River and tributaries of Lake Buhi	Buhi	Camarines Sur	inland	3	5	15	0
30/3/2023	05.14−05.44	south tributary of Lake Buhi	Buhi	Camarines Sur	inland	0.5	4	2	0
30/3/2023	18.30−19.30	Barangay Mayaopayawan	Milaor	Camarines Sur	inland	1	6	6	0
30/3/2023	19.30−22.30	Barangay Mayaopayawan	Milaor	Camarines Sur	inland	3	6	18	0
1/4/2023	19.00−21.00	Barangay Balogo	Pasacao	Camarines Sur	coastal	1	6	6	4
2/4/2023	16.00−16.30	Barangay Caranan, mangroves	Pasacao	Camarines Sur	coastal	0.5	6	3	0
2/4/2023	18.30−19.45	Sitio Suminabang, Barangay Dalupaon	Pasacao	Camarines Sur	coastal	6	1.25	7.5	0
2/4/2023	21.20−22.00	Barangay Balogo	Pasacao	Camarines Sur	coastal	0.66	6	3.96	0
8/6/2023	19.00	Barangay Manapao, from fish pond while electric fishing	Minalabac	Camarines Sur	inland	1	1	1	3
13/6/2023	18.30−19.10	Barangay Mananao, edge of fish pond (tilapia), in mangrove area ~700 m from ocean	Tinambac	Camarines Sur	coastal	0.66	6	3.96	13
15/6/2023	18.10−19.44	Barangay Manapao, fish ponds	Minalabac	Camarines Sur	inland	0.57	6	3.42	0
16/6/2023	19.00	Barangay Manapao, in fish pond in mud	Minalabac	Camarines Sur	inland	1	1	1	9
16/6/2023	18.50−19.45	Sitio Mainit, Barangay Balogo, in stream connected to ocean, 50 m from beach	Pasacao	Camarines Sur	coastal	0.92	6	5.52	9
17/6/2023	19.30−20.30	Barangay Sogod, in freshwater stream from Mt Malinao, in water column, ~1100 ft from river mouth	Tiwi	Albay	coastal	1	4	4	4

We collected voucher specimens by hand, photographed representative specimens of populations and subsequently recorded their body mass and length measurements. Specimens were euthanized humanely (approved University of Kansas Institutional Animal Care and Use Committee protocol authorization 158-04 to R.M.B.) and preserved according to standardized methods [30–32]. Prior informed consent (PIC) documentation was obtained from all communities (municipalities), and issued to J.M.B. Specimen collection, transportation and export complied with all legal permits, guidelines and restrictions by the relevant local and national Philippine authorities (DENR-BMB Wildlife Gratuitous Permit (GP) to collect biological species numbers 258, 270, 292 and 324 to RMB/KU and GP R5-155 issued to MCL/PSU), and all research activities were conducted under the aegis of multiple Memorandum of Agreement among the primary collaborating institutions (DENR, ADNU, PSU and KU), with particular attention to equitability, safety and inclusive fieldwork principles, agreed upon by all parties [33]. Identification of *Cerberus* was based on Murphy [20] and Murphy *et al.* [34]. All collected individuals were deposited in the University of Kansas (KU) Biodiversity Institute and Natural History Museum (voucher specimens KU 351823−351828; 352171−352224).

### 2.3. Study taxa

*Cerberus schneiderii* and *C. microlepis*, the two homalopsid snake species native to The Philippines, differ substantially in their biogeographic and systematic characteristics [35]. *Cerberus schneiderii*, the Southeast Asian bockadam, has one of the widest distributions of any vertebrate, ranging from the eastern edges of the Malay Peninsula, throughout Indochina, The Philippines, Greater and Lesser Sunda Islands and eastern Indonesian Islands, as far east as the Maluku Islands (figure 1). *Cerberus schneiderii* is found throughout all Philippine PAICs [36,37] and likely on all large islands with suitable habitat. Although *C. schneiderii* has been reported from freshwater systems in rivers, lakes, streams and rice paddy fields, it is considered to be a mangrove/coastal, brackish water specialist [20,34]. The widespread and (primarily) coastal distribution of *C. schneiderii* stands in stark contrast to the Philippine micro-endemic species *C. microlepis*, the Lake Buhi bockadam, which is considered to only inhabit this 18 km^2^ freshwater lake in Camarines Sur. Lake Buhi is isolated from the surrounding landscape, with Mt Asog (1196 m elevation) to the west of the lake, and mountainous terrain as high as ~800 m elevation surrounding all other edges of the lake. Although *C. schneiderii* and *C. microlepis* are phenotypically quite similar, *C. microlepis* has a higher number of mid-body dorsal scale rows (27−31, usually 29 versus 21−27, usually 23 in *C. schneiderii* [34]). Although previous studies [13,34,38,39] lightly discussed the taxonomy of *C. microlepis*, we refrain from any taxonomic inference in this work, because a systematic treatment is outside the scope of our niche modelling approach (see below). In this article, we nominally refer to all populations of *C. schneiderii* and *C. microlepis*, together, by just using the genus *Cerberus*, and refer to the Lake Buhi population as *C. microlepis*.

### 2.4. Tests for sexual dimorphism

During our collection efforts, we obtained the mass, snout–vent length (SVL), tail length (TL), total length (TtL) and tail-to-total length ratio (TL:TtL) for each specimen. For specimens for which we had information on sex, we performed Student’s *t*-tests to identify if the means of each of these variables were significantly different between males and females. *F*-ratio tests were used to determine if the variance between arrays was significantly different or not. Raw morphological data can be found in electronic supplementary material, table S1.

### 2.5. Ecological niche modelling

Because our focus is to report new distribution and natural history data for *Cerberus* in The Philippines for conservation efforts and assess local importance of snake fauna in downstream studies, we employed a niche modelling approach to identify suitable habitats of *Cerberus* in The Philippines. Prior molecular studies that include *C. microlepis* from Lake Buhi, Philippines, and *Cerberus dunsoni* from Palau, have identified these taxa as likely conspecifics of *C. schneiderii*. Thus, we run our ecological niche models combining occurrence records of *C. schneiderii*, *C. microlepis* and *C. dunsoni* into one dataset to maximize the environmental variation that is encapsulated by these taxa. Nonetheless, we still use the nominal taxa designations to keep in accordance with the current taxonomy of the group and identify the populations discussed herein (*C. microlepis* = Lake Buhi, Camarines Sur, Philippines; *C. dunsoni* = Palau; *C. schneiderii* = all Philippine populations outside Lake Buhi). Because *Cerberus* is known to occur throughout all major island groups of The Philippines, we use occurrence records to create a niche model projected to The Philippines to understand these snakes’ distribution in the country, including areas that show high habitat suitability but have not been surveyed. We obtained geographic coordinates for a total of 219 samples from public databases (e.g. GBIF) and our field-collected data. For records from GBIF, we downloaded human observation and natural history records that have coordinated localities, and filtered the dataset to remove erroneous localities (e.g. records in the ocean; records representing the closely related *Cerberus rynchops*). We used Maxent v. 3.4.3 [40,41] and a custom pipeline in R [42] that uses the following packages: rJava [43], dismo [44], dplyr [45], ecospat [46], ENMeval [47], ggplot2 [48], maptools [49], maps [50], raster [51], rasterVis [52], RColorBrewer [53], rgdal [54], sf [55], spThin [56], tidyverse [57] and viridis [58]. We used the 19 bioclimatic variables from WorldClim2 [59] at a 2.5 min resolution as predictor variables. We removed all layers that were correlated using a Pearson correlation with a threshold value of 0.80. The remaining bioclimatic variables used were:

BIO1 = annual mean temperature; BIO2 = mean diurnal range (mean of monthly (max. temperature – min. temperature)); BIO4 = temperature seasonality (standard deviation × 100); BIO7 = temperature annual range (max. temperature of warmest month – min. temperature of coldest month); BIO12 = annual precipitation; BIO15 = precipitation seasonality (coefficient of variation); BIO16 = precipitation of wettest quarter; BIO18 = precipitation of warmest quarter; and BIO19 = precipitation of coldest quarter.

Species-specific parameter tuning was performed using ENMeval v. 2.0 [47] using five different feature class combinations: L, LQ, H, LQH and LQHP (L = linear; Q = quadratic; H = hinge; P = product), and regularization multiplier values of 1 through 5. The best combination of feature classes was chosen based on the model with the lowest ΔAIC_c_ value (features = LQ; rm = 1). Models of climate and habitat suitability were projected using the present uncorrelated bioclimatic variables.

### 2.6. Linear models and significance tests

To determine if *Cerberus* are statistically more likely to be found near mangroves and coasts (coasts here defined as coastline, whether mangroves are present or not), we ran linear models, ANOVA and Student’s *t*-tests on our habitat suitability data and the distance of specimen occurrence points from mangroves and from coastlines. We extracted habitat suitability values for *Cerberus* occurrence records from output raster files from ecological niche models (see §2.5). We measured the distance of each record to the closest coastline using Philippines shape files in QGIS v. 3.4.3 Madeira, and also measured the distance of records to the closest mangrove cell using the shapefile of mangrove data (2020) from the Global Mangrove Watch v. 3.0 dataset [60]. We ran linear regression models to determine if habitat suitability was significantly correlated with (i) distance of occurrence records to the closest mangroves (*km* ^*2*^ *man*) and (ii) distance of occurrence records to the closest coastline (*km^2^coast*). Linear regressions were run using the *lm* function in R [61]. Linear models were run with both variables using the equation *habitat suitability ~ km^2^man + km^2^coast*. We also ran an ANOVA to determine if habitat suitability was significantly different between three categories of habitat based on distance from mangroves: coastal (within a mangrove cell or 21 km from mangroves), inland–coastal (1−5 km from mangroves) and inland (>5 km from mangroves). This tested for significant differences in habitat suitability for the following: (i) inland versus inland–coastal; (ii) inland versus coastal; and (iii) inland–coastal versus coastal. We followed our ANOVA with a Tukey honest significant differences (TukeyHSD) test to identify which comparisons were significantly different. Additionally, we ran a Student’s *t*‐test to compare habitat suitability between inland versus inland–coastal + coastal localities. We also ran linear models to determine if salinity (PPT) is correlated with habitat suitability at occurrence points from our niche model. All analyses were run after removing two outlier points of *Cerberus* that were found more than 40 km from the coast (all other samples found within 23 km of the coast).

## 3. Results

### 3.1. *Cerberus* encounters

**Figure 2.**
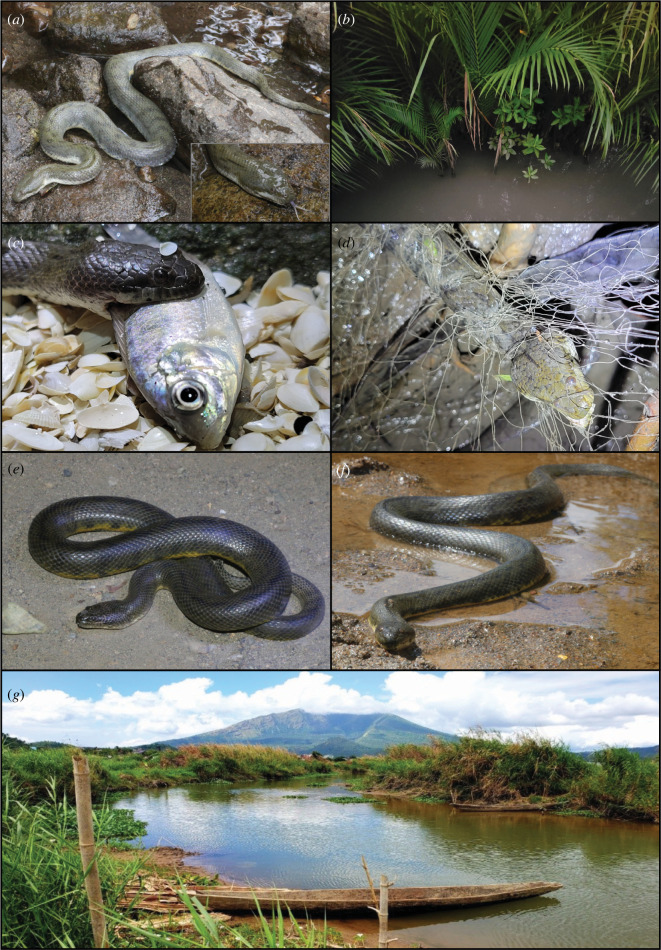
*Cerberus schneiderii* and *C. microlepis* found in Camarines Sur, Philippines. (*a*) Female *C. schneiderii* (voucher specimen KU 352173) from Barangay Balongay, Municipality of Calabanga. Inset photo of head is from the same individual. (*b*) Mangrove habitat in Barangay Balongay where *Cerberus* were found. (*c*) A male *C. schneiderii* (KU 352180) feeding on a *Gerres* sp. fish from the Libmanan River side of the dike in Barangay Balongay. (*d*) Live *C. schneiderii* stuck in discarded fishing net on mangrove side of dike. (*e*,*f*) *C. microlepis* from Lake Buhi. (*g*) Freshwater stream entering Lake Buhi, with Mt Asog in the background.

Our field expeditions resulted in a total of 141 h of active search time (1041 person-hours; table 1). Our expeditions through the BRB were focused on detecting *Cerberus*, whereas the CP expeditions were general collecting efforts. On the CP, our survey efforts resulted in a total of 1 *C. schneiderii* in 34 days (~95 h, ~812 person-hours), which was found at an inland–coastal locality. All other CP searches were performed at inland–coastal localities, and one costal locality, but no *Cerberus* were encountered. The BRB surveys spanned over a total of 28 days (~46 h, ~229 person-hours), in which we found 69 *C*. *schneiderii* and 6 *C*. *microlepis* (figure 2). Of these, 55, 2 and 18 were from coastal, inland–coastal and inland localities (6 of which represent *C. microlepis* from Lake Buhi). All snakes were found at low elevations (<50 m above sea level (a.s.l.)), including Lake Buhi at ~90 m a.s.l.). Our encounters for *C. schneiderii* represent the first municipal-level records of this species in Camarines Sur (Municipalities of Calabanga, Magarao, Minalabac, Pasacao and Tinambac) and Albay (Municipality of Tiwi).

### 3.2. Natural history observations and water measurements

We provide natural history observations for Philppine *C. schneiderii* and *C. microlepis. Cerberus schneiderii* was recorded syntopically with two species of snakes, three lizards and seven frogs (table 2). We documented *C. schneiderii* from a variety of aquatic habitats, including coastal brackish mangroves and mangrove/nipa palms (*Nypa fruticans*), freshwater streams, fish farms (salt water) and fish ponds (fresh water). In contrast, *C. microlepis* was only observed in freshwater streams, approx. ≤30.5 m from the river mouth, where they flowed into Lake Buhi.

**Table 2.** List of taxa which were found in the same locality as *C. schneiderii* during surveys from 2019 to 2023.

family	taxon	locality
Acrochordidae	*Acrochordus granulatus*	Barangay Ponong, Municipality of Magarao, Camarines Sur Province Barangay Sogod, Municipality of Tiwi, Albay Province
Colubridae	*Boiga angulata*	Barangay Santa Cruz, Municipality of Caramoan, Camarines Sur Province
Agamidae	*Bronchocela marmorata*	Barangay Santa Cruz, Municipality of Caramoan, Camarines Sur Province
Agamidae	*Draco spilopterus*	Barangay Santa Cruz, Municipality of Caramoan, Camarines Sur Province
Scincidae	*Lamprolepis smaragdina*	Barangay Santa Cruz, Municipality of Caramoan, Camarines Sur Province
Dicroglossidae	*Fejervarya moodiei*	Barangay Balongay, Municipality of Calabanga, Camarines Sur Province
Dicroglossidae	*Limnonectes woodworthi*	Barangay Balogo, Municipality of Pasacao, Camarines Sur Province Barangay Santa Cruz, Municipality of Caramoan, Camarines Sur Province
Dicroglossidae	*Occidozyga laevis*	Barangay Santa Cruz, Municipality of Caramoan, Camarines Sur Province
Ceratobatrachidae	*Platymantis dorsalis*	Barangay Balogo, Municipality of Pasacao, Camarines Sur Province Barangay Santa Cruz, Municipality of Caramoan, Camarines Sur Province
Rhacophoridae	*Polypedates leucomystax*	Barangay Santa Cruz, Municipality of Caramoan, Camarines Sur Province
Ranidae	*Sanguirana luzonensis*	Barangay Santa Cruz, Municipality of Caramoan, Camarines Sur Province
Ranidae	*Hylarana erythraea*	Barangay Balongay, Municipality of Calabanga, Camarines Sur Province

*Cerberus schneiderii* was found inhabiting waters with a wide range of salinities, of 1−30 PPT, and in water temperatures ranging from 25.6°C to 29.8°C (table 3). We recorded notable differences in how frequently *Cerberus* were found at coastal, inland–coastal and inland localities. We detected 18 *Cerberus* at inland localities over 31.6 search hours (178.4 person-hours), with only 3 of 16 nights resulting in finding *Cerberus*. At inland–coastal localities, two *Cerberus* were encountered during a total of 92.8 search hours (784.3 person-hours), with only 2 of 34 nights resulting in encountering *Cerberus*. Finally, in coastal localities, we found 55 *Cerberus* over 17.7 h (81.9 person-hours), in which *Cerberus* were found during 6 out of 12 nights. At some of these coastal localities, dozens of *Cerberus* individuals can be seen within minutes of searching.

**Table 3.** Water measurements for six localities in Camarines Sur: salinity (PPT), specific gravity and temperature (°C). Data readings were taken three times (M1, M2 and M3) and averaged. Water measurements were recorded three times in Barangay Balongay due to missing measurements on first survey. Microhabitat information of localities at which *Cerberus* were encountered can be found in table 1.

water measurement	M1	M2	M3	average	locality	date	*Cerberus* encountered?
salinity (PPT)	3	1	NA	2	Brgy. Balongay (mangrove side), Munic. Calabanga, Camarines Sur Prov.	17/3/2023	yes
specific gravity	NA	NA	NA	NA	Brgy. Balongay (mangrove side), Munic. Calabanga, Camarines Sur Prov.	17/3/2023	yes
temperature (°C)	25.6	25.8	NA	25.7	Brgy. Balongay (mangrove side), Munic. Calabanga, Camarines Sur Prov.	17/3/2023	yes
salinity (PPT)	3	4	4	3.67	Brgy. Balongay (Libmanan River side), Munic. Calabanga, Camarines Sur Prov.	18/3/2023	yes
specific gravity	1.003	1.003	1.003	1.003	Brgy. Balongay (Libmanan River side), Munic. Calabanga, Camarines Sur Prov.	18/3/2023	yes
temperature (°C)	29.1	28.7	28.6	28.8	Brgy. Balongay (Libmanan River side), Munic. Calabanga, Camarines Sur Prov.	18/3/2023	yes
salinity (PPT)	3	3	3	3	Brgy. Balongay (mangrove side), Munic. Calabanga, Camarines Sur Prov.	18/3/2023	yes
specific gravity	1.002	1.002	1.002	1.002	Brgy. Balongay (mangrove side), Munic. Calabanga, Camarines Sur Prov.	18/3/2023	yes
temperature (°C)	28.7	28.3	28.1	28.37	Brgy. Balongay (mangrove side), Munic. Calabanga, Camarines Sur Prov.	18/3/2023	yes
salinity (PPT)	1	1	1	1	Brgy. Balogo, Munic. Pasacao, Camarines Sur Prov.	1/4/2023	yes
specific gravity	1.001	1.001	1.001	1.001	Brgy. Balogo, Munic. Pasacao, Camarines Sur Prov.	1/4/2023	yes
temperature (°C)	29.8	29.8	29.8	29.8	Brgy. Balogo, Munic. Pasacao, Camarines Sur Prov.	1/4/2023	yes
salinity (PPT)	30	30	30	30	Brgy. Mananao, Munic. Tinambac, Camarines Sur Prov.	13/6/2023	yes
specific gravity	1.022	1.022	1.022	1.022	Brgy. Mananao, Munic. Tinambac, Camarines Sur Prov.	13/6/2023	yes
temperature (°C)	29	29	29	29	Brgy. Mananao, Munic. Tinambac, Camarines Sur Prov.	13/6/2023	yes
salinity (PPT)	0	0	0	0	Brgy. Sogod, Munic. of Tiwi, Albay Prov.	13/6/2023	yes
specific gravity	1	1	1	1	Brgy. Sogod, Munic. of Tiwi, Albay Prov.	13/6/2023	yes
temperature (°C)	NA	NA	NA	NA	Brgy. Sogod, Munic. of Tiwi, Albay Prov.	13/6/2023	yes
salinity (PPT)	0	0	0	0	Lake Bato, Munic. Bato, Camarines Sur Prov.	23/3/2023	no
specific gravity	1	1	1	1	Lake Bato, Munic. Bato, Camarines Sur Prov.	23/3/2023	no
temperature (°C)	28.1	28.1	28.1	28.1	Lake Bato, Munic. Bato, Camarines Sur Prov.	23/3/2023	no
salinity (PPT)	0	0	0	0	Lake Buhi, Munic. Buhi, Camarines Sur Prov.	29/3/2023	no
specific gravity	1	1	1	1	Lake Buhi, Munic. Buhi, Camarines Sur Prov.	29/3/2023	no
temperature (°C)	30.8	30.8	30.8	30.8	Lake Buhi, Munic. Buhi, Camarines Sur Prov.	29/3/2023	no

During our surveys, we recorded multiple natural history observations. We discovered a male individual (KU 352186) of *C. schneiderii* feeding on a goby in Barangay Balogo, Municipality of Pasacao in Camarines Sur. We limit the taxonomic identification of the prey item to a member of the family Oxudercidae, but it is likely *Boleophthalmus* cf. *gracilis*. In Barangay Balongay, Municipality of Calabanga, Camarines Sur, we found a male *C. schneiderii* (KU 352180) eating actinopterygian fish, *Gerres* sp. (figure 2), and another individual (not collected) consuming the dicroglossid frog *F. moodiei*. We also observed sidewinding behaviour from *C. schneiderii* in Barangay Balongay and *C. microlepis* at Lake Buhi, when individuals on land (i.e. not in streams or flooded mangroves) attempted to escape upon the approach of biologists. One individual of *C. microlepis* demonstrated a threat display in which it coiled its body and teetered back and forth on one of its coils. After a few seconds, it flipped its tail upside down revealing a yellow and block-mottled venter, and then sidewinded to escape.

Our analyses on morphological data (28 females; 27 males) revealed that SVL was significantly higher in females than in males (*F =* 0.152; *p =* 0.010). We also found that males had significantly longer tales (TL:TtL) than females (*F =* 0.892; *p =* 0.001). No significant differences were observed in TL (*F =* 6.54 × 10^−8^; *p =* 0.590) or TtL (*F =* 0.028; *p =* 0.072), separately, between males and females.

### 3.3. Ecological niche and linear models

**Figure 3.**
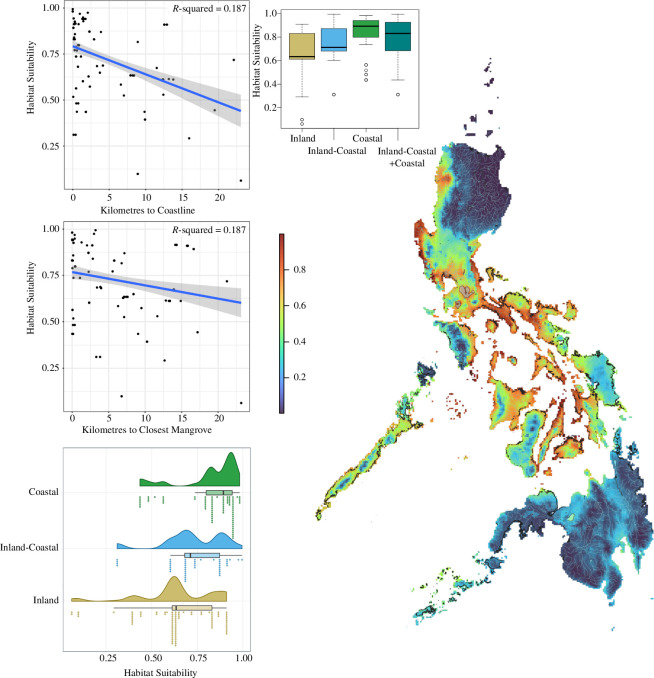
Ecological niche model and statistical analyses for *Cerberus* occurrence records and environmental data. Linear models show relationship of habitat suitability of occurrence records in the niche model to distance of records to the nearest coastal line (top) or mangrove (middle). Bottom rainfall plot shows density of habitat suitability for coastal, inland–coastal and inland habitats. Boxplots show distribution of habitat suitability for each of these categories, as well as grouping inland–coastal and coastal into one category. Ecological niche model (right) shows habitat suitability, with lower and higher suitability represented by cooler and warmer colours, respectively (scale bar). Black edges around Philippines niche model are the environmental 2020 mangrove data layer.

Our niche models show the highest habitat suitability in low-elevation areas near coastlines and mangrove habitats (area under the curve (AUC) = 0.832; Boyce index (BI) = 0.931; figure 3). The lowest suitability values (0.002) were seen in northern Luzon and the Mindanao PAIC. Central and southern Luzon, the West Visayan Islands and Palawan all show intermediate to high (~0.50−1.0) suitability along the coastlines and lower elevational inland areas. Intermediate- and high-elevation areas (e.g. Mts Malinao, Asog, Mayon and Isarog in Camarines Sur) show lower suitability (~0.25−0.16).

Our linear models with both predictor variables (*km^2^man* and *km^2^coast*) showed that habitat suitability of *Cerberus* is significantly correlated with both distance from coasts (*p =* 6.51 × 10^−9^) and distance from mangroves (*p =* 0.026) (multiple *R* ^2^ = 0.187; *F*[2,214] = 24.58). Additionally, we found significant differences between habitat types (ANOVA: *F*[2,214] = 23.18; *p* = 7.76 × 10^−10^; figure 3). A TukeyHSD showed that habitat suitability is higher in coastal habitats than inland–coastal habitats (adjusted *p =* 0.017), higher in coastal than inland habitats (adjusted *p =* 0.000) and higher in inland–coastal than inland habitats (adjusted *p =* 0.011). Inland–coastal + coastal categories also had higher suitability than inland localities (Student’s *t*‐test: *p* = 2.689 × 10^−6^). We found that habitat suitability had no correlation with PPT at measured localities (*R* ^2^ = 0.049; *F*[1,5] = 0.2568; *p* = 0.6339).

## 4. Discussion

Natural history observations and online repositories that provide geological and environmental data can fill in gaps in research aiming to determine microhabitat preference and distributions of species [62,63]. In this study, we take an inclusive approach to curating all possible data from field collection efforts for *C. schneiderii* and *C. microlepis* (e.g. date and time of collection, natural history observations, microhabitat records, water measurements and proximity of collection localities to other habitat types). Detailed recording of these occurrences allowed us to provide the first statistical evidence, at least for Philippines populations, for *Cerberus* preference towards mangrove and coastal habitats. Freshwater inland lacustrine habitat, exemplified by Lake Buhi, exemplifies *C. microlepis* [34], but inland freshwater habitats also characterizes *C. schneiderii* populations, as seen during our own efforts and reported by others [24].

We provide the first detailed report of *C. schneiderii* microhabitats and several natural history observations. Our study provides the first assessment of sexual dimorphism in *Cerberus* in The Philippines. Although our own field sampling is limited to one province (Camarines Sur, localities outside Lake Buhi), we find results that are consistent with sexual size dimorphism measures of populations in West Java [25]. Additionally, while *Cerberus* are considered piscivorous [64], we document the first instance of any *Cerberus* feeding on a frog (*F. moodiei*). When encountered, approached and attempted to capture the snake, it released the deceased, partially digested frog. We assume that our observation constitutes an instance of interrupted scavenging, but we are unable to rule out an earlier predation event. At the locality where this was observed (Barangay Balongay, Calabanga), a concrete dike separates the Libmanan River, that empties into San Miguel Bay, from the mangroves that border the barangay. The two dietary records from this locality (instances of snakes feeding on fish versus a snake feeding on the frog) were recorded on either side of this artificial habitat boundary. We only found frogs on the mangrove side of the dike, but noted availability of riverine fish on the Libmanan River side. We find it possible that *Cerberus* has a more generalized diet [65] than previously assumed, and includes both fish and amphibians (J. Murphy 2023, personal communication). Additionally, we found that many females were gravid (movement of neonates can be observed externally from the venter) and found neonates (e.g. KU 352186) in mangroves, feeding on gobies during our July 2023 expedition [65], but none were observed in our March 2023 surveys.

Although *C. schneiderii* has been reported broadly from throughout The Philippines, a conspicuous sampling gap has persisted within Camarines Sur. Our fieldwork provides 66 new occurrence records from BRB and CP (figure 3), filling in a major distributional gap on Luzon [35]. Our survey efforts were less successful at inland localities, and we recorded fewer occurrences away from coasts than we did more successfully in the vicinity of coastal mangroves and estuaries. Nevertheless, in Camarines Sur, we did substantiate *Cerberus* occurrences at inland localities, albeit only on 11 (of 62) days. *Cerberus* were only observed on 3 of 16 days searching inland areas. Despite some localities having plenty of aquatic habitat with similar salinity to other *Cerberus*-heavy areas, edge habitat and abundant prey (e.g. tilapia fingerlings and adults, frogs), we found no *Cerberus* at the inland localities of Lakes Bato and Baao. We also found no *Cerberus* at Lake Buhi in 2023, despite credible reports, a week after our survey included. It is possible that the population at Lake Buhi represents a population of *C. schneiderii* that may have been historically isolated by the substantial elevational topographic relief surrounding this lake. Although second-hand information may be subject to erroneous identifications and other subjective bias, we received accounts from >10 different inland locations of *Cerberus* being much more ‘common’ (versus ‘rare’ now) in the past (which, if accurate, could be due to over exploitation), or resident impressions of snakes apparently extirpated since the construction of concrete dikes for flood control. Although we have no comparative data that allow us to test whether flood control construction has negatively affected *Cerberus* populations, we note that these concrete structures (typically 5−7 m tall, with walls perpendicular to the water level), leave no edge habitat for *Cerberus* to rest, feed or reproduce. Additionally, in coastal areas where we encountered discarded fishing nets, we consistently recorded dead and live entangled *Cerberus* (figure 2); *Cerberus* that were freed from the net and got back into it were stuck in it again within seconds. Coastal areas support abundant populations of these snakes when there is adequate mangrove and nipa palm habitat, and even modified habitats such as fishponds. Fishponds of adequate sizes with plenty of fish that are adjacent to suitable habitat like mangroves (e.g. Tinambac) seem to be able to hold hundreds of *Cerberus*. In these areas, *Cerberus* are seen as pests due to their high densities and predation of commercial fish like tilapia.

Interestingly, we found higher numbers of *Cerberus* at inland localities than inland–coastal areas. Such pattern of encounter frequency in the extremes (with regard to our habitat types based on mangroves) could reflect population densities rather than area of occurrence. *Cerberus* had higher densities in small areas on coastlines, and some fishponds at inland localities may represent opportune spots for *Cerberus* to gather in regions of habitat that are not optimal. Although we find *Cerberus* in greater numbers on coasts and at inland localities, we find greater habitat suitability statistics at coastal and inland–coastal habitats, where we also find the highest population densities based on our surveys. We find *Cerberus* consistently at night, with the highest chances of success being during low-tide periods when there is more access to mudflats (and when fish are in shallow water or on the mud (e.g. mudskippers)).

Our observations of high densities of *Cerberus* in coastal systems are reflected in the results from our niche models and statistical analyses. Niche models show *Cerberus* have much higher habitat suitability at low elevation, coastal areas (figure 3). Indeed, this includes the coasts of the CP. Contrary to what would be expected based on the high habitat suitability shown in the niche model, we only found 1 *Cerberus* in 34 total survey days. Many factors can influence species distributions outside of bioclimatic variables and mangroves, such as competition, abiotic environmental factors and varying levels of human disturbance due to agricultural fields or habitat degradation. Thus, while we are confident in the results of our niche model, continued surveys in the CP will reveal how abundant *Cerberus* is in this part of The Philippines.

Although our model predicted low habitat suitability in northern Luzon and Mindanao (and northern Palawan) compared to estimates from southern Luzon and the West Visayan islands, we suspect that this pattern is artefactual, and likely due to a lack of occurrence records from the northern and southern portions of the archipelago. There have been confirmed sightings of *Cerberus* during fieldwork in Mindanao, and we suspect that future field surveys will identify additional occurrences beyond estimates of suitability provided here through modelling efforts. Based on the salinity and observation data we have collected, *Cerberus* can likely tolerate a wide range of salinities (0−30 PPT), and although we have limited locations with salinity measurements, there is no significant correlation between PPT and suitability. Nevertheless, based on our linear models and empirical observations reported here, suggesting the rarity of inland *Cerberus*, it appears mangrove habitat is likely critical for *Cerberus* population health. Given the high densities of *Cerberus* and their dietary preferences for fish and amphibians, aquatic mud snakes may be an important aspect of food dynamics and ecosystem health in mangrove systems, which are one of the most important environmental systems for biodiversity and provide critical ecosystem services [66,67]. This study serves as an example of how detailed species occurrences, and microhabitat observations can enhance our knowledge of species-specific natural history to obtain a broader understanding of species distributions and their roles in the respective ecosystem.

## Data Availability

All data, code for analyses and respective input files are available on Github [68], and have been archived within the Zenodo repository [69]. All precise locality data are available online from Specify hosted by the University of Kansas (KU) Biodiversity Institute and Natural History Museum. The morphological dataset supporting this article has been uploaded as part of the electronic supplementary material [70] and is also available on Github [68].
